# Visual Processing Matters in Chinese Reading Acquisition and Early Mathematics

**DOI:** 10.3389/fpsyg.2020.00462

**Published:** 2020-04-01

**Authors:** Xiujie Yang, Xiangzhi Meng

**Affiliations:** ^1^Faculty of Psychology, Beijing Normal University, Beijing, China; ^2^School of Psychological and Cognitive Sciences, Beijing Key Laboratory of Behavioral and Mental Health, Peking University, Beijing, China; ^3^PekingU-PolyU Center for Child Development and Learning, Beijing, China

**Keywords:** visual processing, Chinese reading, mathematics performance, kindergarten children, longitudinal study

## Abstract

The main purpose of the present study was to investigate whether visual processing uniquely contributed to character reading and early mathematics in Chinese children. Eighty-two Chinese kindergarteners at K3 (mean age = 68 months, *SD* = 0.30) were followed up to grade one (mean age = 82 months, *SD* = 0.35) with an interval of 14 months. Nonverbal intelligence, inhibitory control, sustained attention, character reading, and mathematics were measured at kindergarten. Character reading and mathematics were assessed again at grade one. Results showed visual processing at kindergarten significantly predicted character reading at grade one after controlling for prior reading performance, inhibitory control, sustained attention, age, gender, and nonverbal IQ. Similarly, visual processing at kindergarten explained unique variance in early mathematics at grade one when prior mathematics performance and other covariates at kindergarten were controlled. These findings suggest that visual processing should serve as a domain-general precursor of children’s performance in character reading and early mathematics and an important cognitive factor for later academic learning.

## Introduction

Visual processing is the procedure of organizing and interpreting visual information (Siok and Fletcher, 2001; McGrew, 2009). It reflects the ability of attending to and distinguishing a figure’s features and details, such as shape, orientation, color, and size (Kulp et al., 2004; Yang et al., 2013). In the past decades, evidence has shown that poor performance in visual processing might explain learning difficulties in reading and mathematics (Barrett, 1965; O’Neill and Stanley, 1976; Corballis et al., 1985; Boden and Brodeur, 1999). For example, children who have deficits in visual perception often cannot efficiently distinguish words printed in different sizes or colors (Fischer et al., 2000; Visser et al., 2004). Students suffering from visual perception impairment usually exhibit reversals and misaligning of horizontal series of digits, and weak math visualization (Willows et al., 2012). People with visual processing deficits are often diagnosed with dyslexia (e.g., Talcott et al., 2000; Amitay et al., 2002; Ramus et al., 2003) and/or dyscalculia (O’hare et al., 1991; Emmons and Anderson, 2005).

Reading and mathematics abilities are important for future academic success (Jordan et al., 2009; Cooper et al., 2014). Besides, there are at least three additional reasons for investigating the effect of visual processing on reading acquisition and early mathematics. First, previous researchers have found high overlaps between visual skills and domain-general cognitive skills (e.g., sustained attention, nonverbal reasoning, and inhibitory control) (Buckley et al., 2018; Yang et al., 2019a), as well as RAN (Yang et al., 2019b). However, it remains unclear whether visual processing will uniquely contribute to reading and mathematics when these general cognitive abilities are taken into consideration. Second, even among those existing studies, very few provided longitudinal evidence among kindergarten children about the predictive effect of visual processing on their future Chinese reading (e.g., Huang and Hanley, 1995, 1997; Ho, 1997; McBride-Chang et al., 2005) and mathematics (e.g., Zhang and Lin, 2015). Third, as is known, the transition of early reading and early mathematics from kindergarten to primary school could be problematic for children who fall behind in basic cognitive abilities (LeFevre et al., 2010; Gunderson et al., 2012; Yang et al., 2019a). If visual processing can uniquely contribute to reading and mathematics, perhaps educators and parents would pay more attention to children’s visual skills and identify children at risk of learning difficulties as early as possible.

The role of visual skills in early reading development may be stronger for Chinese readers than for readers of alphabetic languages. That is because many Chinese characters are visually similar but are usually pronounced differently. Meanwhile, they convey different meanings, for example, “±” (tu3, means “earth”) and “±” (shi4, means “soldier”). Moreover, Chinese characters also feature nonlinear visual processing; they involve more complex spatial configuration processing, e.g., left–right, up–down, inside–outside, enclosed, semi-enclosed, and within stroke patterns. Szwed et al. (2014) have found that Chinese reading involves enhanced engagement of intermediate visual areas compared to alphabetic reading. Compared to alphabetic readers, Chinese readers tend to be more likely to make use of the visual symbols as the basis of orthographic knowledge in Chinese characters (Luo et al., 2013; Zhou et al., 2015). Metalinguistic skills, such as phonological awareness, morphological awareness, and orthographic knowledge, are found to be associated with visual processing (McBride-Chang et al., 2005; Martens and de Jong, 2006; Chung et al., 2008; Luo et al., 2013). Indeed, Chinese children are found to rapidly distinguish the visual–orthographic characteristics from their similar counterparts and match them with the phonological representations stored in long-term memory (e.g., McBride-Chang et al., 2005; Luo et al., 2013). Otherwise, children might exhibit difficulties in visually distinguishing similar characters because there are more diverse spatial patterns among strokes compared to English (Ding et al., 2002). For example, Chinese primary school children with dyslexia usually struggle to distinguish “
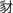
” (jackal) and “
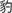
” (leopard) and have difficulty in identifying whether “lonely” should be written as “
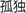
” or “
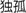
” (the former is correct) (Ding et al., 2002).

In addition, McBride (2016) proposed that Chinese characters’ visual distinctiveness may lead children to foster categorization strategies in recognizing characters (McBride, 2016). Indeed, there has been evidence showing that Chinese beginning learners at the age of 3 acquire character reading abilities using a visual strategy of discriminating the graphic feature in the character, given that Chinese characters are composed of radicals with complicated spatial structures (Ho and Bryant, 1997). Therefore, we believe that visual processing should be more essential in Chinese character recognition. However, among a relatively small body of literature with different conclusions (e.g., Huang and Hanley, 1995, 1997; Ho, 1997; McBride-Chang et al., 2005), most studies were conducted in Hong Kong and Taiwan, where traditional Chinese is used. Mainland Chinese children learn simplified characters, while children in Hong Kong and Taiwan learn traditional Chinese characters (McBride, 2016). The semantic and phonetic radicals in characters facilitate readers’ pronouncing and understanding the meanings of these characters. Simplified characters are more difficult to recognize than traditional characters because the radical clues have been cut down (Cui et al., 2019). Mainland Chinese children have to rely more on visual processing to carefully recognize the remaining radical clues in each character. Based on the findings on traditional Chinese reading (Chen and Yuen, 1991; McBride, 2016; Cui et al., 2019), we hypothesized that visual processing also would be important for simplified Chinese reading.

With regard to early mathematics, we propose that visual processing, along with early numeracy knowledge, such as counting, number sense, and number knowledge (Jordan et al., 2010; Garon-Carrier et al., 2018), and general cognitive skills, such as sustained attention, nonverbal reasoning, and inhibitory control (LeFevre et al., 2010), are essential in its development. To acquire calculation and number comparison skills, individuals need to visually recognize number words and transform number symbols into quantities, at least among young children (Geary, 2004; Krajewski and Schneider, 2009a, b). According to Dehaene’s triple-code model, the visual system is one of the most important systems of number representation, and numbers can be encoded as Arabic numerals (Dehaene, 1992; Dehaene and Cohen, 1995). Indeed, visual skills can facilitate mathematics problem solving by forming an accurate “mental blackboard” to organize the interrelations between numerical quantities (Tartre, 1990; Zhang et al., 2014; Zhang and Lin, 2015). Children with high visual–spatial skills tended to translate more math problems into pictorial and spatial descriptions and demonstrated better flexibility in changing a formed word problem into an arithmetic problem, abstracting arithmetic formulas from applied word problems, and mentally processing number relationships (Talcott et al., 2000; Sigmundsson et al., 2010; Zhang and Lin, 2015). In contrast, children with inadequate visual processing had difficulty in breaking an arithmetic formula into a spatial mental process (Fennema and Tartre, 1985; Zhang et al., 2017). Indeed, children with disabilities in arithmetic exhibited lower visual–spatial abilities (Rourke and Finlayson, 1978; Sigmundsson et al., 2010). Neuroimaging evidence also indicates that proficient math performers tended to make use of spatial-based strategies that are associated with parietal regions (Rivera et al., 2005). Importantly, Tosto et al. (2014) have suggested a strong genetic overlap between mathematical ability and visual–spatial processing, indicating the important role of visual processing in children’s mathematics development.

In contrast to mature math learners who use fact retrieval strategies, kindergarten children often use mental counting to solve arithmetic problems (Geary et al., 1993). They usually transform a mathematics problem into a mental spatial format that represents how numbers are correlated to each other (Yang et al., 2019a). For example, to solve an addition problem (e.g., 5 + 6), children might turn it into a visual finger counting or mental counting process. These abilities of abstracting and classifying characteristics of objects during counting with mental spatial skills usually play important roles in mathematics learning. As such, we hypothesize that visual processing is essential for spatial mental activity and proficient mathematics problem solving, and the development of visual processing would predict later mathematics performance of Chinese children. However, some studies solely considered visual processing as part of intelligence, suggesting that age and verbal ability may moderate the relationships between visual–spatial ability, reading, and mathematics (Huang and Hanley, 1995, 1997; Buckley et al., 2018; Frick, 2018). Moreover, there are relatively few Chinese studies investigating relations of spatial abilities and mathematics development apart from general cognitive abilities (Zhou et al., 2015; Zhang, 2016), especially among mainland Chinese kindergarten children who are beginning to learn mathematics.

### The Present Study

In the past decades, there has been a debate on whether visual processing contributes to reading acquisition (Kavale, 1982; Stanovich, 1992; Huang and Hanley, 1995, 1997; Ho and Bryant, 1997, 1999; Hu and Catts, 1998; Siok and Fletcher, 2001; McBride-Chang and Kail, 2002; McBride-Chang et al., 2005) and mathematics learning (Zhou et al., 2015; Cui et al., 2019). Meanwhile, there is a scarcity of research investigating reading and mathematics simultaneously to identify their common underlying factors among Chinese young children.

The present study intended to investigate the potentially simultaneous role of visual processing in reading acquisition and early mathematics among mainland Chinese young children. We chose children at kindergarten and followed them up to grade one, since this is the period when children go from receiving informal education to formal education, and there was a high possibility to find out those children’s improvements in reading and mathematics performance (Yang et al., 2019a, c). Visual processing was measured by the orientation discrimination task, which distinguishes orientation differences between two separated lines (O’Neill and Stanley, 1976; Wang et al., 2014). The task required rapid visual processing to identify the orientation of the two lines and to perceive whether a line was tilted clockwise or anticlockwise. Spatial orientation has been consistently shown as the one of the most important visual perceptual/processing abilities among all visual–spatial abilities (Silver and Hagin, 1970; O’Neill and Stanley, 1976; Yilmaz, 2009; Wang et al., 2014; Zhang, 2016). People have found that the spatial orientation threshold between the two lines is higher in children with dyslexia than in typically developed children (O’Neill and Stanley, 1976). Therefore, we hypothesized that visual processing would simultaneously contribute to children’s reading and mathematics performance concurrently and longitudinally. Specifically, children who were higher in the orientation discrimination thresholds were expected to achieve lower scores in reading and mathematics.

Some researchers proposed that the contribution of visual processing to reading and mathematics performance could be explained by general cognitive abilities such as intelligence (e.g., Olson and Datta, 2002). More researchers indicated that the effects of visual processing on reading and mathematics still existed even when general cognitive abilities were controlled (Kavale, 1982; Talcott et al., 2000). Therefore, further research is necessary to include other general cognitive abilities to clarify the unique relations of visual processing with reading and mathematics. Considering that visual processing was highly associated and shared much variance with inhibition and sustained attention (Buckley et al., 2018; Yang et al., 2019a), along with general intelligence (Olson and Datta, 2002; Cui et al., 2019), we assessed them with the Flanker task, the Continuous Performance Task (CPT), and the Combined Raven’s Test (CRT). The current study set out to provide evidence for the unique role of visual processing in children’s reading and mathematics acquisitions in their early years, in turn contributing to the screening and remediation of children at risk of dyslexia, dyscalculia, or both.

## Materials and Methods

### Participants

The study began with 108 Chinese kindergarteners (63 boys and 45 girls) who attended their last year at senior kindergarten. They were followed up to grade one after 14 months. Informed consent was obtained from the headmaster and the children’s parents. Ethics approval was sought from the Survey and Behavioral Research Ethics Committee of Peking University. All children had no auditory or visual deficits. Vision or correct vision was normal. The sample size dropped to 82 by the second measurement because some children were not enrolled in the same school or had moved away. Thus, the dropout rate was approximately 24%. We conducted an independent *T*-test and found no significant differences in all measures between the dropout children and the remaining children (all *p* > 0.05). At the beginning of the study, the children’s average age was 5 years and 8 months (*SD* = 0.30); at grade one, the children’s average age was 6 years and 10 months (*SD* = 0.35). All of these children were fluent in Mandarin.

### Measures

#### Character Reading

This measure was revised from a standardized Chinese character recognition test (Wang and Tao, 1996). It involved 142 Chinese characters that were divided into 10 sets based on their levels of difficulty. Children were required to read the Chinese characters aloud when they were in senior kindergarten and grade one. The experimenter recorded the total number of characters that children read correctly. The correct answer was transformed into children’s character reading performance based on the norm.

#### Mathematics

We used calculation and word problems to assess children’s mathematics abilities. The calculation task was adapted from Butterworth (2003) and Rodic et al. (2015), in which 50 simple calculation problems (e.g., 6 + 2) were presented on a computer screen one by one, and the administrator would, meanwhile, orally present them. Children needed to choose between two candidate choices to get the correct answer as quickly and accurately as possible. The total test time was 2 min. To reduce the guessing effect in this timed task, we used the correction formula *S* = *R* − *W*/(*n* − 1) to calculate the total score, where *S* stands for the adjusted score, *R* refers to the number of correct responses, *W* represents the number of incorrect responses, and *n* is the number of alternative responses to each item (Guilford, 1936). In the word problems, children were asked to compare the result of one calculation task with one specific number in some context (Wei et al., 2012). For example, the experimenter stated one story in Chinese: “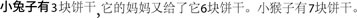 [A rabbit has three cookies, and her mother gave her another six cookies. A monkey has seven cookies].” And then the experimenter asked: “ 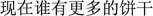 [Now who has more cookies]?” As such, children needed to comprehend the context and compute the involved numbers in order to get the answer. The test was stopped when three consecutive errors were made. The total score is 24, with 1 point for one correctly answered item.

#### Visual Processing

An orientation discrimination task was adopted to assess children’s sensitivity in discriminating orientation differences between two segments (Wang et al., 2014). In the task, children were asked to determine which direction the second segment turned compared to the first segment (clockwise/anticlockwise). Therefore, children needed to understand the representation of the segment and the spatial relations between the two segments. On each trial, the order of being clockwise or anticlockwise was randomly determined. To adapt to children’s ability, we changed the original pure lines and pure dots into a bicolor segment based on the pilot study. This is easier than the original task because children had more cues to judge if the segment shifted clockwise or anticlockwise. The segment was 6° length presented in the first quartile. A three-down-one-up self-adaptive rule was used, and the step size was 1.0715°. That is to say, when the orientation differences between the two segments started from 10.239°, it would decrease 1.0715° when the children answered correctly three times in succession but rose 1.0715° when the children answered incorrectly once.

**FIGURE 1:**
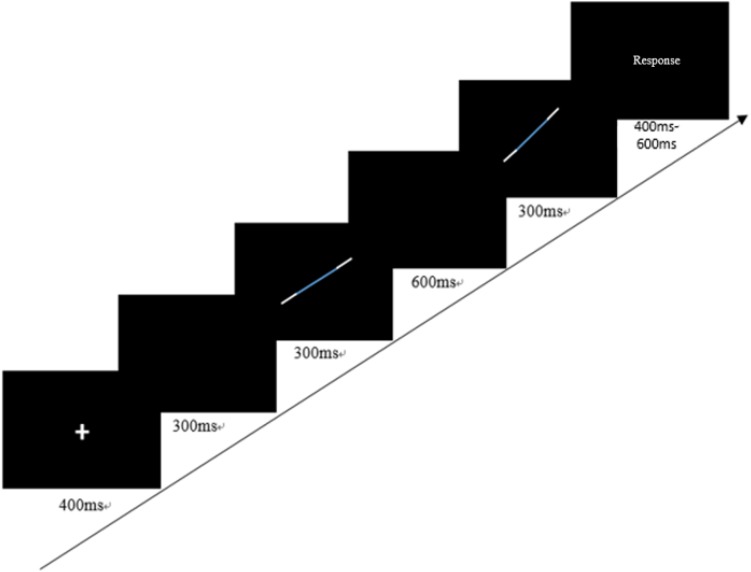
A trial of the orientation discrimination task.

The task had two blocks with 10 reversals in each block. The reversal occurred when the orientation differences changed between the two segments. The orientation discrimination threshold was the indicator of the visual processing task, which was calculated with the mean of the angle differences between the two solid segments. To help children adapt to the task, and to avoid fatigue, we only used the mean of the angle differences in the middle six reversals as the orientation discrimination threshold. The lower the threshold, the higher the visual processing ability. To keep the visual angle consistent, children were required to sit 80 cm away from the central screen. The concrete procedure for each trial is shown in Figure 1.

#### Sustained Attention

**FIGURE 2:**
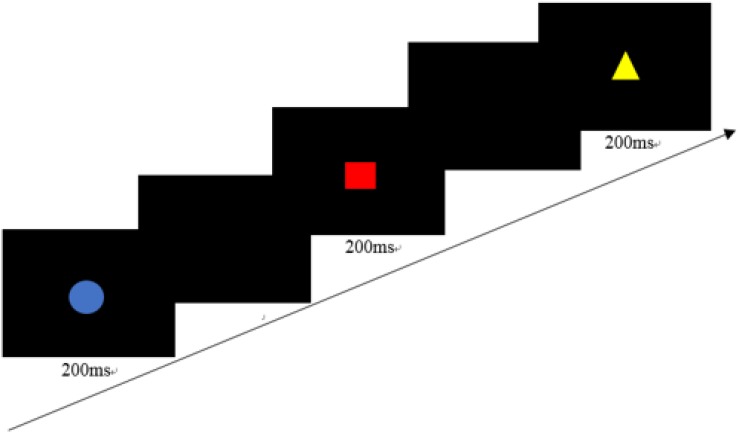
A trial of the Continuous Performance Task (the interval time is 1,500, 2,000, 2,500, or 3,000 ms).

The CPT is typically conceptualized as an assessment of sustained attention (Gardner-Neblett et al., 2014). There were 16 types of figures (□, ○, △, and ✩, colored red, yellow, blue, or green), each presented on the screen in the center of the computer screen for 200 ms one at a time. The interval time between two stimuli was randomly distributed to 1,500, 2,000, 2,500, or 3,000 ms. During the task, the child was required to press the left key when the red square appeared on the screen but ignore other stimuli (Figure 2). Ten practice trials would first appear, followed by 120 test trials.

#### Inhibitory Control

**FIGURE 3:**
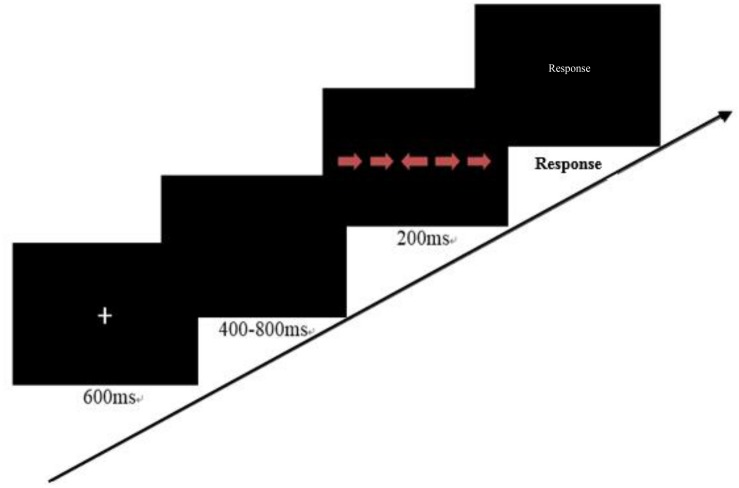
A trial of the Flanker task (there is no time limit for response).

We used an adapted version of the Flanker task to test children’s inhibitory control ability (Ridderinkhof and van der Molen, 1995; Miller and Cohen, 2001). In this task, the child was told a story that a young princess was lost in the forest, and there were some arrow marks to assist her home. Only the arrow laying in the central position is the correct direction. In the incongruent condition, arrows pointed to the direction opposite the central one; in the congruent condition, all of the arrows pointed to the same direction. The instructor asked the child to point out the direction by pressing the left or right key. Ten practice trials were first presented and were followed by 40 experimental trials. The procedure is shown in Figure 3.

#### Nonverbal IQ

The CRT was used to measure children’s nonverbal IQ (Li et al., 1989). This test has 72 matrices with sets A, A_*B*_, B, C, D, and E of increasing difficulty. For set A, children were required to identify the missing element to fill the blanks of an incomplete picture. For the next four sets, children needed to select one element that was suitable in a matrix that met a certain rule. Children had to stop after 20 min, or when they made three consecutive errors in the row of C, D, or E. We converted the total number of matrices solved correctly to a standard score based on the CRT norm from urban China for children aged 5 to 16 years.

### Procedure

Children completed a battery of measures as in the above description along with some others as part of a large-scale longitudinal study. For the kindergarten assessments, children were tested individually on several consecutive days. Nonverbal IQ, sustained attention, inhibitory control, orientation discrimination task, calculation, word problems, and character recognition were conducted. After 14 months, as when children entered grade one, calculation, word problems, and character recognition measures were conducted. Each session was administered in a quiet room at school within 30 min to avoid fatigue.

## Results

### Preliminary Analyses

The means, standard errors, skewness, kurtosis, and reliabilities for all variables assessed at senior kindergarten and grade one are included in Table 1. All data are presented as raw scores. Specifically, sustained attention is presented as reaction time in the CPT in which children responded to the target stimulus, and inhibitory control is presented as reaction time in the Flanker task in the incongruent condition.

**TABLE 1:** Descriptive statistics for variables measured at senior kindergarten and grade one.

	Variables	Measures	Maximum	Mean (SE)	Range	Reliability
Kindergarten	General cognitive abilities	Nonverbal IQ	140	116.55 (1.23)	90.00–140.00	0.75
		Sustained attention	–	639.06 (9.24)	449.45–946.89	0.85
		Inhibitory control	–	1221.92 (77.15)	444.31–5,412.48	0.83
	Visual processing	Orientation discrimination task	–	7.51 (0.69)	2.32–27.46	0.79
	Mathematics	Calculation	49.00	15.40 (0.80)	0–49.00	0.89
		Word problems	24.00	14.67 (0.73)	1.00–24.00	0.99
	Character reading	Character recognition	1006.18	371.82 (33.67)	12.12–997.48	0.99
Grade one		Calculation	49.00	25.83 (1.01)	7.00–49.00	0.90
		Word problems	24.00	20.26 (0.57)	7.00–24.00	0.99
	Character reading	Character recognition	1006.18	743.01 (29.33)	99.31–1,006.18	0.99

*The range in sustained attention and inhibitory control means reaction time (ms).*

In order to investigate how children developed from kindergarten to grade one, we had their mathematics (calculation and word problems) and character reading abilities as the dependent variables with a paired-sample *T*-test. Results showed that, for character reading, *t*(1, 80) = 12.81, *p* < 0.001; for calculation, *t*(1, 72) = 10.79, *p* < 0.001; for word problems, *t*(1, 75) = 6.60, *p* < 0.001. Therefore, children had significant improvements in mathematics and character reading from kindergarten to grade one. Because we had multiple measures for early numerical processing and mathematics, we used principal component analysis to create factor scores to index early numerical processing (rapid digit naming, number identification, and numerical reasoning) and mathematics (calculation and word problems).

Table 2 displays the correlations among visual processing and general cognitive abilities at kindergarten, character reading, and mathematics at kindergarten and grade one. Considering that the reaction time and accuracy in the sustained attention task were correlated with each other (trade-off effect: *r* = −0.33, *p* < 0.01), we performed a formula S = RT × (1 + 2 × error) to correct the result, in which RT stands for correct reaction time in the hit condition and error indicates the false negative alarm rate (children did not respond when the target appeared, when they should have). The formula has been widely used before to handle trade-off measures (e.g., Lyons et al., 2014). Similarly, for inhibitory control, the Flanker task also demonstrated a trade-off effect (*r* = −0.31, *p* < 0.01), and we employed the same formula dealing with the problem. In the Flanker task, RT and error were from the incongruent condition. Table 2 demonstrates that visual processing was significantly related with mathematics and character reading either at senior kindergarten or at grade one.

**TABLE 2:** Correlations among all variables at kindergarten and grade one.

Variables	1	2	3	4	5	6	7	8	9	10
1. T1 nonverbal IQ	—									
2. T1 inhibitory control	−0.32***	—								
3. T1 sustained attention	−0.25**	0.41***	—							
4. T1 visual processing	−0.24*	0.10	0.17	—						
5. T1 character reading	0.30**	–0.11	−0.23*	−0.23*	—					
6. T1 calculation	0.35***	–0.19	−0.23*	−0.24**	0.35***	—				
7. T1 word problems	0.36***	−0.30**	−0.24*	−0.23*	0.27**	0.55***	—			
8. T2 word problems	0.22	–0.02	−0.32**	−0.35**	0.06	0.20	0.26*	—		
9. T2 calculation	0.36**	–0.13	–0.21	−0.24*	0.42***	0.51***	0.21	0.21	—	
10. T2 character reading	0.33**	–0.13	–0.18	−0.32**	0.76***	0.38***	0.26*	0.17	0.39***	—

T1, senior kindergarten; T2, grade one. ***p < 0.001; **p < 0.01; *p < 0.05.

### Regression Analyses

To further investigate how visual processing was associated with character reading, we performed hierarchical regression for character reading and mathematics, controlling for gender, age, nonverbal IQ, sustained attention, and inhibitory control. *R*^2^, *R*^2^ change, standardized beta coefficients, and *t* scores are reported in Table 3 for character reading and mathematics, respectively. In order to estimate the risk of multicollinearity, we checked the *tolerance* and the *variance inflation factor* (*VIF*) values of the predictors calculationally. In this analysis, the *tolerance* values were all larger than 0.50, and *VIF* values were all smaller than 2.0, indicating that there was no multicollinearity in the current analysis. Table 3 demonstrates that, when gender and age were statistically controlled, general cognitive factors, including nonverbal IQ, attention, and inhibitory control, significantly explained 18% of the variance in character reading. Interestingly, after initial character reading at senior kindergarten was further controlled, visual processing still significantly explained 4% of the variance in character reading. As regards mathematics, we conducted univariate hierarchical regression analysis to see whether visual processing would account for it. Table 3 indicates that, when gender and age were statistically controlled, general cognitive factors significantly explained 17% of the variance in mathematics. Interestingly, after initial mathematics at senior kindergarten was further controlled, visual processing still significantly explained 7% of the variance in mathematics. The *tolerance* values were all larger than 0.54, and *VIF* values were all smaller than 1.80, indicating that there was no multicollinearity in the current analysis. We also controlled metalinguistic awareness in order to spell out their effects (see Supplementary Table S1).

**TABLE 3:** Hierarchical regression analysis of first grade character reading with visual processing, general cognitive abilities, and initial character reading controlled.

Steps	Measures	T2 character reading							T2 mathematics					
		*R*^2^	Δ*R*^2^	*Beta*	*t*	*Tolerance*	*VIF*		*R*^2^	Δ*R*^2^	*Beta*	*t*	*Tolerance*	*VIF*
1	Age	0.03	0.03	–0.02	–0.21	0.88	1.14		0.00	0.00	–0.05	–0.47	0.85	1.77
	Gender			0.07	0.86	0.94	1.06				–0.18	–1.57	0.88	1.14
2	T1 character reading/T1 mathematics	0.56	0.54***	0.7	8.33***	0.83	1.2		0.2	0.20***	0.37	2.60***	0.57	1.77
3	T1 nonverbal IQ	0.58	0.02	0.09	0.97	0.71	1.41		0.25	0.05	0.02	0.17	0.61	1.63
	T1 sustained attention			0.03	0.35	0.73	1.36				–0.15	–1.23	0.79	1.27
	T1 inhibitory control			0.02	0.27	0.74	1.36				0.18	1.5	0.82	1.22
4	T1 visual processing	0.62	0.04*	–0.20	−2.49*	0.87	1.15		0.32	0.07*	–0.30	−2.54*	0.82	1.22

T1, senior kindergarten; T2, grade one; VIF, variance inflation factor. ***p < 0.001; **p < 0.01; *p < 0.05.

Overall, after all general cognitive factors, age, gender, and initial reading and mathematics performance were controlled, visual processing at senior kindergarten still significantly accounted for the variance in character reading and mathematics at grade one.

## Discussion

In the present study, we investigated whether and how visual processing predicted young Chinese children’s reading and mathematics performance, including character reading and mathematics abilities. To achieve this goal, we tested kindergarten children’s visual processing, reading, and mathematics performance, and followed up 14 months later. We found that visual processing was concurrently and longitudinally associated with character reading and early mathematics. To rule out possible confounding factors, we also assessed three important cognitive skills, including intelligence, attention, and inhibitory control. Results showed that visual processing still significantly explained variance in future character reading and mathematics abilities, even when children’s age, gender, these general cognitive abilities, initial reading, and initial mathematics performance were controlled for.

Visual processing is a required step for discriminating letters, characters, symbols, and written numbers (McBride-Chang et al., 2005; Meng et al., 2011). As prior work showed, all visual reading and mathematics development should involve basic visual processing (Zhou et al., 2015). In line with the arguments from alphabetic languages (Franceschini et al., 2012, 2013), to recognize characters, Chinese children need to develop a visual strategy of discriminating the visual graphic features and their relations within the character (Ho and Bryant, 1997). For example, the Chinese characters “±” (means “earth”) and “±” (means “solider”) are quite similar visually. When seeing the two characters clearly, one will visually notice that their difference lies in whether the upper horizontal line in the characters is longer than the lower horizontal line. Many children with reading disabilities often show difficulties in distinguishing one from the other. For example, the spatial orientation difference threshold between the two straight lines is higher in children with dyslexia than in typically developed children (O’Neill and Stanley, 1976). This is also consistent with English studies that showed that the left fusiform visual word form area explained the maximum effect of mirror priming (e.g., “b” vs. “d”) in children with reading disabilities (Dehaene et al., 2010).

Furthermore, visual processing shapes the way visual information is extracted from print (Aghababian and Nazir, 2000). Specifically, visual processing might be important for Chinese children to reduce an indecipherable character into simple character radicals (McBride-Chang et al., 2005; Luo et al., 2013). For example, the Chinese character “
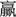
” (means “win”) can be visually divided into five Chinese characters (“
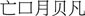
”), making it easier for young children to recognize the character. As we have argued, the Chinese writing system is heavily orthographically based, which is different from English, which is phonologically based (e.g., Chen and Kao, 2002; Shu et al., 2003; Hsiao and Shillcock, 2006). Chinese children with higher visual processing exhibit greater ease at acquiring the visual–orthographic knowledge in characters, which in turn facilitates character recognition (Siok and Fletcher, 2001). In other words, during reading, the quality of the visual–orthographic form mapped onto the lexical representation could affect children’s performance (Perfetti and Hart, 2002). Although previous literature has indicated conflicting relations of reading and visual processing among children in Taiwan and Hong Kong (e.g., Huang and Hanley, 1995, 1997; Ho, 1997), mainland kindergartners who spoke Mandarin and learned the simplified script usually developed stronger visual discrimination skills than their counterparts in Hong Kong (Peng et al., 2010; Luo et al., 2013) and demonstrated stronger relations of visual processing with Chinese character reading (McBride-Chang et al., 2005).

Similarly, the current findings support that visual processing promotes children’s development of mathematics skills. The present study examined calculation via addition and subtraction and word problems via story problem solving. Visual processing expertise may contribute to gains in these mathematics subskills through the following ways. First, visual processing may influence an individual’s calculation ability through his/her capacity of representing number words and obtaining quantity information (Simon et al., 1998). Supporting evidence is that, when children learn to count objects, they need to distinguish the quantity from the visual features of each object (e.g., color, size). For example, to count apples one by one, young children need to visually figure out the corresponding quantity of each apple setting aside the volume or color of each apple. Second, visual perception reflects children’s understanding, recognizing, and interpreting relationships between object stimuli. In order to fulfill the orientation discrimination of the present study, children needed to distinguish the location of each segment and the spatial relations between the two segments; this is similar to solving math problems orally presented, and children needed to represent the addition and subtraction problem and manipulate the numerals mentally. Third, visual processing is particularly important in accurately distinguishing quantities and representing relations between number words mentally (Dehaene and Cohen, 1995; Kulp et al., 2004). In addition to calculation, word problem solving requires children to mentally present the numerals and quantity information to figure out which of the two numbers is larger. Visual processing might therefore support the representations of quantity information via a mental number line (Dehaene, 2003) and might help to form and hold mental representations of the numerals (Tartre, 1990; Geary et al., 1993; Robert and LeFevre, 2013). Consistent with this claim, Gunderson et al. (2012) revealed that children’s visual–spatial skill predicted their performance on calculation and that this relation was mediated by children’s knowledge of linear number line estimation. In sum, visual processing is necessary for young children to mentally represent number words from calculation problems and story problems, do calculations mentally, and compare quantities in story problems mentally (Fennema and Tartre, 1985; Assel et al., 2003; Landerl et al., 2004; Vaidya, 2004; Sigmundsson et al., 2010).

The current study demonstrated insignificant correlations between inhibitory control and reading and early mathematics (except for word problems at kindergarten). This might be because the cognitive workload of our assessments was relatively low. For example, we assessed single character reading rather than long words, and simple addition rather than four arithmetic operations. More reading and mathematics measures should be included to investigate how measure types might moderate the associations of visual processing with reading and mathematics. In addition, although the results provide relatively straightforward evidence for the association among visual processing, character reading, and mathematics performance, intervention studies to investigate the causality of visual processing in reading and mathematics are still needed. Moreover, the orientation discrimination task administered in this study is only one kind of visual discrimination, but visual processing demonstrates various abilities to identify features and details of stimuli, including shape, orientation (the present study), color, and size (Ho and Bryant, 1999; Kulp et al., 2004; Rohde, 2008). Therefore, it is important for future studies to include various visual processing components and test their associations with Chinese character reading. Finally, longitudinal studies examining visual processing, reading, and mathematics at multiple time points are needed given that there might be a bidirectional association between visual processing and reading acquisition in the early years (e.g., Goswami, 2015). Reading Chinese might even show a stronger effect on visual skills than reading some alphabetic languages (Demetriou et al., 2005). The association of visual processing with mathematics was similar to that with reading (e.g., Sigmundsson et al., 2010).

Altogether, this research is among the first to follow Chinese children from kindergarten to first grade and to investigate whether and to what extent visual processing predicted later reading and mathematics competence. Particularly, we included both Chinese character reading and early mathematics, which are the most important foundations of children’s later academic success. Our findings demonstrated that visual processing served as a domain-general indicator that contributed to organizing information to help solve different types of academic problems, in spite of nonverbal intelligence, inhibitory control, and attention. The present results showed potential implications for education and practice. Specifically, educators, psychologists, and parents should pay more attention to how visual processing might affect children’s early learning (Basch, 2011). For example, standardized assessments of visual processing can be developed in the future to screen children at risk of reading and mathematics difficulties. Moreover, systematic visual processing training, including spatial relations, visual perceptions, and orientation discriminations, along with Chinese radicals and written numbers, might be conducted among children to assist them in learning characters and early mathematics knowledge.

## Data Availability Statement

The datasets generated for this study are available on request to the corresponding author.

## Ethics Statement

The studies involving human participants were reviewed and approved by the Ethics Committee of School of Psychology, Peking University. Written informed consent to participate in this study was provided by the participants’ legal guardian/next of kin.

## Author Contributions

XY designed the experiments, collected, and analyzed the data, and wrote the manuscript. XM designed the experiments interface, discussed the data analyses, and commented on the manuscript. Both authors gave the final approval of the version to be published.

## Conflict of Interest

The authors declare that the research was conducted in the absence of any commercial or financial relationships that could be construed as a potential conflict of interest.
