# Supplementary material for: Visual Processing Matters in Chinese Reading Acquisition and Early Mathematics
Source: Front Psychol. 2020 Apr 1;11:462. doi: 10.3389/fpsyg.2020.00462 (PMC7141237; doi:10.3389/fpsyg.2020.00462)
Supplement: Supplementary file 1 [file Table_1.DOCX]

Table s1. Hierarchical regression analysis of children’s character reading at grade one with visual processing, general cognitive abilities, metalinguistic awareness, and initial character reading controlled.

| Steps | Measures | T2 Character reading | | | |  |  |  |
| --- | --- | --- | --- | --- | --- | --- | --- | --- |
|  |  | *R^2^* | △*R^2^* | *Beta* | *t* | *Tolerance* | *VIF* |  |
| 1  2  3 | Age  Gender  T1Character reading  T1Nonverbal IQ | 0.03  0.57  0.60 | 0.03  0.54***  0.03 | -0.03  0.08  0.71  0.07 | -0.38  0.94  7.12***  0.74 | 0.86  0.89  0.58  0.64 | 1.16  1.13  1.72  1.56 |  |
|  | T1Sustained attention  T1Inhibitory control  T1Phonological awareness  T1Morphological awareness  T1Orthographic awareness |  |  | 0.06  0.03  -.01  .15  -.07 | 0.65  0.37  -.13  1.71  -.76 | 0.71  0.70  0.62  0.76  0.61 | 1.41  1.42  1.61  1.32  1.64 |  |
| 4 | T1Visual processing | 0.64 | 0.04* | -0.20 | -2.60* | 0.85 | 1.18 |  |

Note：T1=senior kindergarten；T2=grade one. *** *p* < 0.001；** *p* < 0.01；* *p* < 0.05.
